# Clinical Observations on Some New Pharmaceutical Preparations

**Published:** 1891-04

**Authors:** 


### CLINICAL OBSERVATIONS ON SOME NEW PHARMACEUTICAL PREPARATIONS.

In a paper read before the Thirty-fourth Quarterly Meeting of the North Central Ohio Medical Society, held at Mansfield, Ohio, September 26, 1890, Dr. R. Harvey Reed, of Mansfield, says:

"Every age in medicine and surgery has had its fanatics, who seemed to live for little else except to ride some particular hobby to death; whilst, on the other hand, every age has had its old fogies who would rather perish than turn an inch to the right or left of the old time-worn rut of their forefathers.

"The hundreds of worthless 'new remedies' that are placed before the profession for their patronage from year to year, is enough to disgust them with all new remedies. It seems to me that many of our manufacturing chemists spend the bulk of their time seeking for something that is new, regardless of its real merits or value.

"If only they can strike the profession with a 'new remedy' of some description or other, they are perfectly happy.

"But with all these criticisms we must admit there is now and then a new remedy comes to light which has real and lasting merit, which in a large degree atones for the defects of many of its worthless compeers."

Then after referring most favorably to the non-irritating preparation of cascara sagrada, prepared by Mr. J. Le Roy Webber, Ph. G., the author makes the following statement as to his experience with pancrobilin:

"In this direction, however, we have another 'new remedy' which has gradually engrafted itself into my good graces, which is becoming more and more permanent the longer I use it. This is what is known as 'pancrobilin' and it is a combination of pancreatin and bile, and placed upon the market in form of a liquid and a pill, of which two I consider the latter more preferable.

"In cases where there is a diminished quantity, or even an absence, of these natural products, especially the bile, resulting in the distressing complication of intestinal or duodenal indigestion, I have found this preparation of decided value by assisting the intestinal digestion until the normal functions of the liver and pancreas, but especially the former, could be established.

"In constipation attended with flatulence, the result of an inactive liver, I have found this remedy of great value, promptly relieving the flatulence, and producing natural cclored stools of a normal consistency, in place of the pale ash colored fæces, or the dry, hard scybala, of the chronic dyspeptic.

"After a careful trial of some three years in a variety of cases affected with constipation resulting from congestion of the liver, and in cases in which there is an atonic condition of the coats of the bowels resulting in intestinal indigestion, I am frank to say that I know of no two remedies that will give as prompt relief to these conditions as the ones under consideration.

"In the one class of cases the pancrobilin supplies the intestine with an artificial supply of bile and pancreatin, which digests the food that otherwise would not be digested, thus giving relief until the real difficulty with the liver can be overcome. In the other class of cases the cascara sagrada tones up the intestine, increases the secretions, which in turn facilitate digestion, and relieves the constipation."—*The American Lancet.*

